# Structure-Based Discovery of Negative Allosteric Modulators of the Metabotropic Glutamate Receptor 5

**DOI:** 10.1021/acschembio.2c00234

**Published:** 2022-09-23

**Authors:** Stefanie Kampen, David Rodríguez, Morten Jørgensen, Monika Kruszyk-Kujawa, Xinyan Huang, Michael Collins, Noel Boyle, Damien Maurel, Axel Rudling, Guillaume Lebon, Jens Carlsson

**Affiliations:** † Science for Life Laboratory, Department of Cell and Molecular Biology, Uppsala University, SE-751 24 Uppsala, Sweden; ‡ Science for Life Laboratory, Department of Biochemistry and Biophysics, Stockholm University, SE-171 21 Solna, Sweden; § H. Lundbeck A/S, Ottiliavej 9, DK-2500 Valby, Denmark; ∥ Lundbeck Research USA, 215 College Road, Paramus, New Jersey 07652 - 1431, United States; ⊥ IGF, Université de Montpellier, CNRS, INSERM, 34094 Montpellier, France

## Abstract

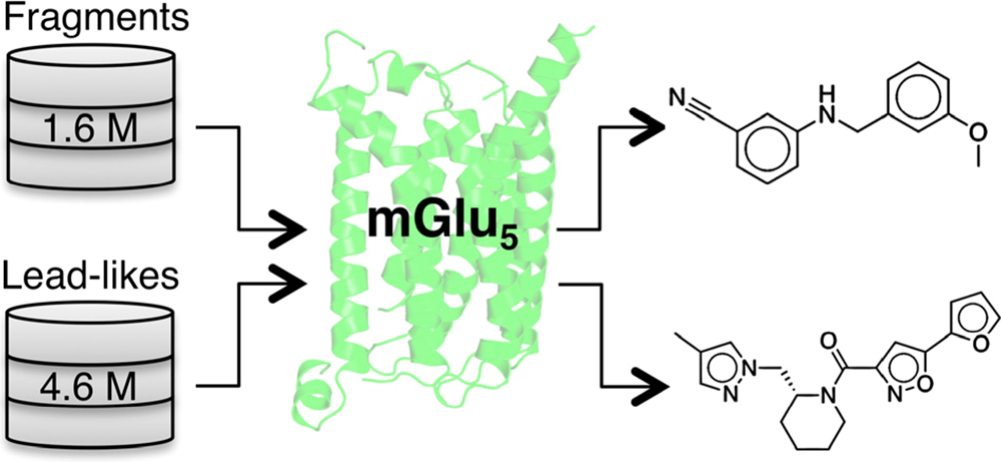

Recently determined structures of class C G protein-coupled receptors (GPCRs) revealed the location of allosteric binding sites and opened new opportunities for the discovery of novel modulators. In this work, molecular docking screens for allosteric modulators targeting the metabotropic glutamate receptor 5 (mGlu_5_) were performed. The mGlu_5_ receptor is activated by the main excitatory neurotransmitter of the nervous central system, L-glutamate, and mGlu_5_ receptor activity can be allosterically modulated by negative or positive allosteric modulators. The mGlu_5_ receptor is a promising target for the treatment of psychiatric and neurodegenerative diseases, and several allosteric modulators of this GPCR have been evaluated in clinical trials. Chemical libraries containing fragment- (1.6 million molecules) and lead-like (4.6 million molecules) compounds were docked to an allosteric binding site of mGlu_5_ identified in X-ray crystal structures. Among the top-ranked compounds, 59 fragments and 59 lead-like compounds were selected for experimental evaluation. Of these, four fragment- and seven lead-like compounds were confirmed to bind to the allosteric site with affinities ranging from 0.43 to 8.6 μM, corresponding to a hit rate of 9%. The four compounds with the highest affinities were demonstrated to be negative allosteric modulators of mGlu_5_ signaling in functional assays. The results demonstrate that virtual screens of fragment- and lead-like chemical libraries have complementary advantages and illustrate how access to high-resolution structures of GPCRs in complex with allosteric modulators can accelerate lead discovery.

## Introduction

G protein-coupled receptors (GPCRs) constitute the largest family of membrane proteins and are expressed throughout the human body. GPCRs play essential roles in cellular communication and regulate numerous signaling pathways that are targets for drug discovery. Approximately one third of all Food and Drug Administration-approved drugs interact with GPCRs, and more than 300 agents targeting these receptors are currently being evaluated in clinical trials.^1^ The majority of the approved drugs are likely to bind to the same pocket as the endogenous ligand (the orthosteric site) and either activate or block receptor signaling. An alternative approach to develop drugs is to focus on allosteric modulators, which by definition bind to sites that are distinct from the orthosteric pocket. Negative allosteric modulators (NAMs) decrease the effect of the ligand bound to the orthosteric site, whereas positive allosteric modulators (PAMs) augment the efficacy or affinity of the orthosteric ligand.^2^ Development of allosteric modulators has the potential to address several challenges in GPCR drug discovery. Allosteric binding pockets are generally less conserved than the orthosteric site, and hence it may be possible to identify compounds with high subtype selectivity, which reduces the risk of drug side effects because of off-target interactions. Moreover, NAMs and PAMs will only modulate receptor activity in the presence of the endogenous agonist, which is not possible with orthosteric ligands and enables more specific control of tissue response.^2−4^ However, the development of allosteric modulators is generally difficult because, in contrast to orthosteric ligands, such compounds cannot be designed based on the endogenous signaling molecule, which has been a successful strategy to identify GPCR drugs.

Advances in X-ray crystallography and cryo-electron microscopy have led to the determination of a large number of high-resolution GPCR structures in complex with ligands and intracellular effectors.^5^ The structures have provided valuable insights into the molecular basis of receptor activation and ligand recognition. A majority of the GPCR structures have been determined in complex with orthosteric ligands, enabling the design of agonists and antagonists using structure-based modeling.^6^ More recently, several structures of GPCRs in complex with allosteric modulators have been determined, revealing that such ligands can occupy diverse pockets in the extracellular loops, the transmembrane region, G protein binding site, and extrahelical pockets facing the lipid membrane.^7^ As the sites of action were largely unknown prior to this structural information, these complexes represent a major breakthrough for understanding the molecular basis of allosteric modulation and structure-based drug design.

**Figure 1:**
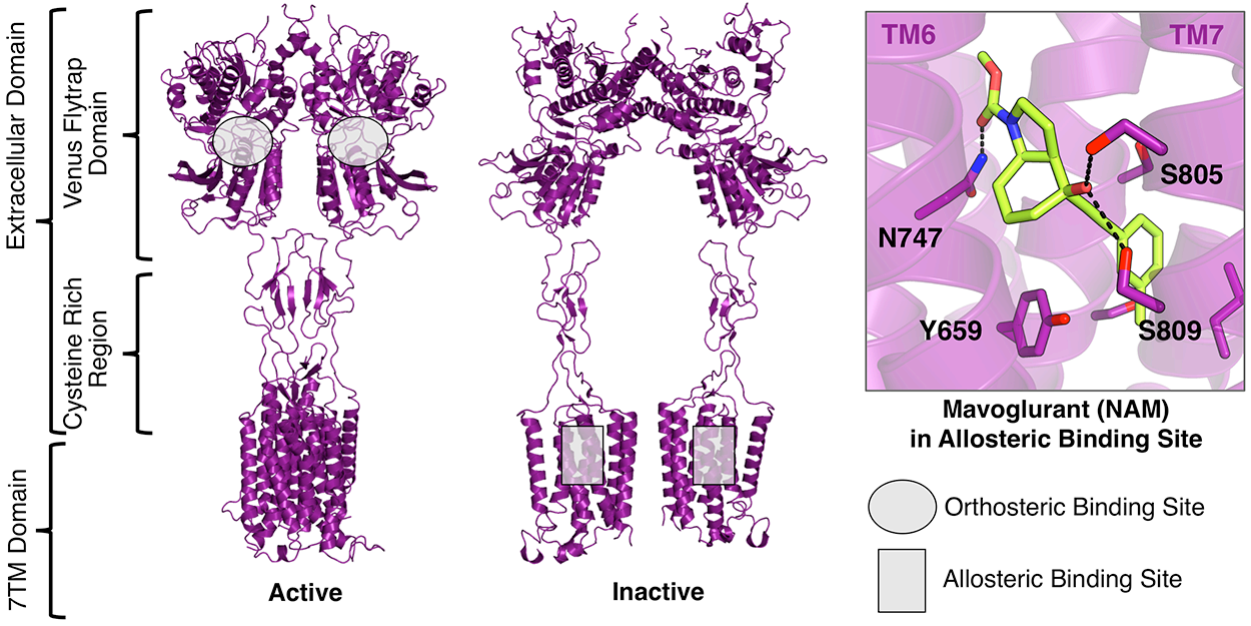
Structure and binding sites of mGlu_5_. The mGlu_5_ receptor consists of an extracellular domain (ECD) (Venus flytrap domain and a cysteine-rich region) and a 7TM domain. Upon agonist binding to the Venus flytrap domain, conformational changes are induced in the 7TM domain that activates G protein signaling (PDB accession code of agonist-bound state: 6N51^8^). Negative allosteric modulators such as Mavoglurant (PDB accession code: 4OO9^9^) bind to a pocket inside the 7TM bundle (PDB accession code of inactive state: 6N52^8^).

**Figure 2:**
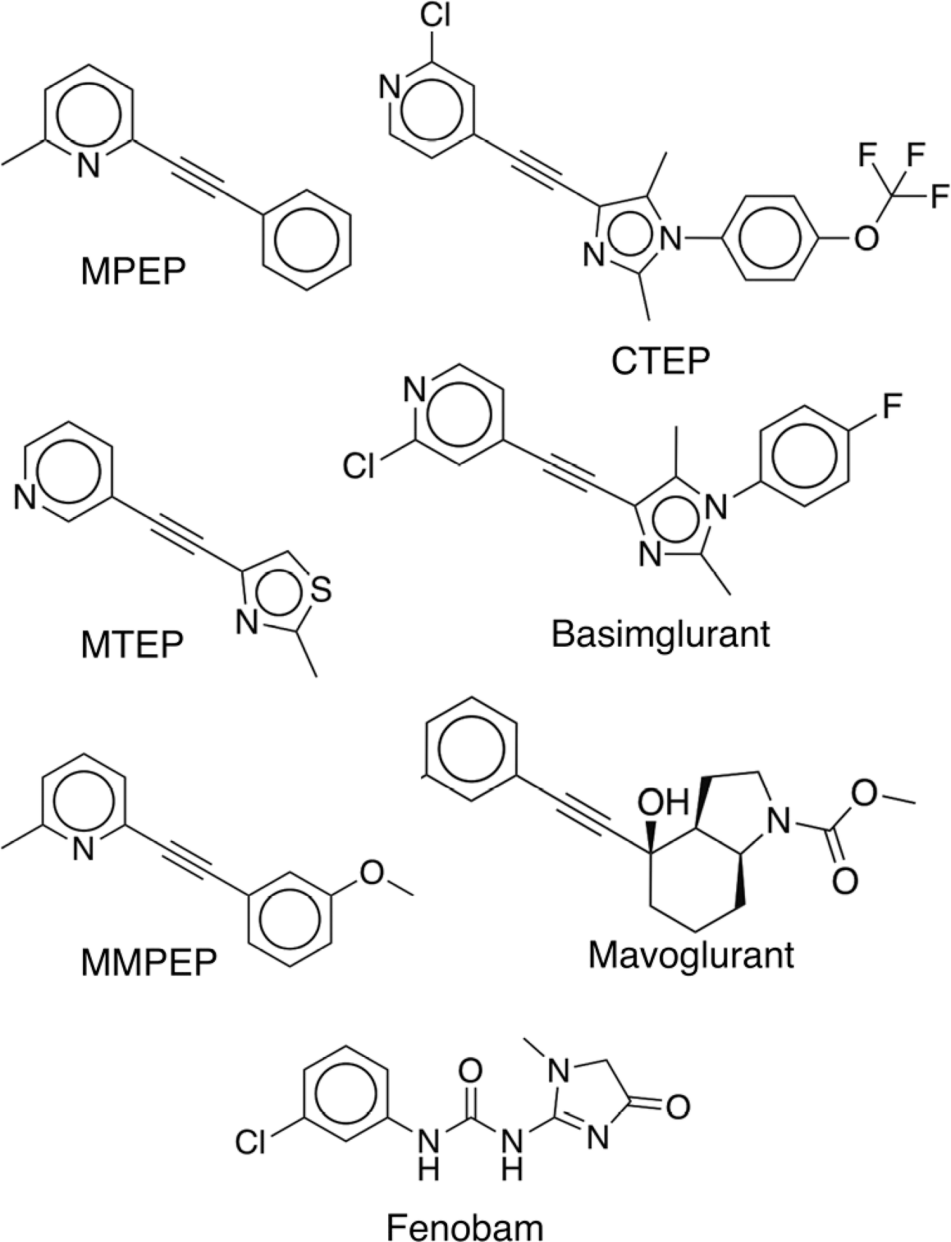
NAMs of mGlu_5_. Examples of mGlu_5_ NAMs (MPEP,^21^ CTEP,^22^ MTEP,^23^ Basimglurant,^24^ MMPEP,^25^ Mavoglurant,^26^ and Fenobam^27^).

Metabotropic glutamate receptors (mGlus) are involved in the regulation of neuronal excitability and synaptic transmission throughout the central nervous system,^10^ and these GPCRs have great potential as drug targets for psychiatric^11^ and neurodegenerative^12,13^ diseases. The eight mGlu subtypes belong to the group of class C receptors, which have several unique structural features. The receptors are only functionally active in their dimeric state, and each monomer has a large ECD at the N-terminal, which contains the Venus flytrap and cysteine-rich domain. The Venus flytrap domain binds the neurotransmitter glutamate and is connected to the heptahelical transmembrane (7TM) domain via the cysteine-rich domain (Figure 1).^10,14−16^ Recently determined structures of the mGlu_5_ subtype show that agonist binding to the ECD leads to conformational changes that stabilize intermolecular interactions between helices in the 7TM domains, which enables G protein coupling (Figure 1).^8,17^ As the glutamate binding site is highly conserved, the development of selective orthosteric ligands has been challenging, and a large number of drug discovery efforts have instead focused on allosteric modulators. Potent NAMs and PAMs of several subtypes have been identified, and mGlu_5_ modulators (Figure 2) have reached clinical trials for different indications (e.g. fragile X syndrome, depression, and Parkinson’s disease).^18^ Crystal structures of mGlu_5_ also revealed that NAMs bind to an intrahelical pocket in the 7TM region and provide opportunities to design novel allosteric modulators using structure-based modeling.^8,9,17,19,20^

In this work, we carried out structure-based virtual screening to assess if allosteric modulators of GPCRs could be identified by molecular docking. Two approaches to identify ligands occupying the NAM binding site of mGlu_5_ were explored by docking of commercial chemical libraries with either fragment- or lead-like compounds. Based on screens of 6.2 million compounds, 118 top-ranked compounds were selected for experimental evaluation in binding assays. Among these, 11 allosteric ligands were identified, and the most potent compounds acted as NAMs in functional assays. The impact of the choice of library on the virtual screening results, comparisons to experimental high-throughput screening campaigns, and the feasibility of virtual screens for allosteric modulators of GPCRs will be discussed.

## Results and Discussion

### Molecular Docking Screening for Allosteric Modulators of mGlu_5_

The structure-based virtual screen focused on an allosteric site identified in a high-resolution crystal structure of mGlu_5_^9^ in complex with Mavoglurant (PDB accession code: 4OO9).^28^ Mavoglurant binds in a deeply buried pocket located in the TM region of the receptor (Figure 1), which has been confirmed to be the interaction site of several other NAMs (e.g. Fenobam and MMPEP,^20^Figure 2), and forms hydrogen bonds with Ser805^7.35×36^, Ser809^7.39×40^, and Asn747^5.47×47^ (superscripts represent GPCRdb numbering^29^). The ability of virtual screening to identify allosteric modulators was evaluated by docking of known mGlu_5_ NAMs and property-matched decoys^30^ to the binding site using the program DOCK3.6.^31−33^ The results of ligand enrichment calculations will depend on the choice of receptor structure and the selection of actives and decoys.^34^ A good ligand enrichment does not guarantee that a prospective virtual screen will be successful, but these control calculations can be useful in the optimization of docking parameters.^35,36^ The enrichment of NAMs was quantified using receiver operating characteristic (ROC) curves, which were used to calculate the adjusted LogAUC values and the ROC-based enrichment factor at 1% (EF_1_, Precent of the ligands identified when 1% of the decoys have been found). Random enrichment corresponds to an adjusted LogAUC value of zero and an EF_1_ of 1, whereas large positive values indicate that there is an enrichment of ligands over decoys.^34^ In the optimization of the receptor structure for virtual screening, different rotamer positions of polar hydrogens in the binding site for six residues were explored (Ser654^3.39×39^, Ser658^3.43×43^, Ser805^7.35×36^, Ser809^7.39×40^, and Tyr659^3.44×44^). A combination of two receptor models, which had different hydroxyl rotamer positions for Ser809^7.39×40^, resulted in good enrichment of known NAMs. This model had an adjusted LogAUC of 28 and an EF_1_ of 23, which indicated a strong enrichment of NAMs by molecular docking screening (Supporting Information Figure S1).

In the prospective virtual screen, two different chemical libraries from the ZINC12 database^37^ were docked to the allosteric site. The first library contained 1.6 million fragment-like compounds (*M*W ≤ 250 Da) and was selected because many potent mGlu_5_ NAMs are of similar size (e.g., MPEP, *M*W = 193 Da). The second library contained 4.6 million lead-like compounds (250 Da < *M*W < 350 Da), and these larger molecules had the potential to form additional interactions in the binding site compared to fragments. Each compound was docked in thousands of orientations and up to several hundred conformations. Binding energies were predicted using a physics-based scoring function.^31−33^ For each library, the compounds were ranked based on docking energy, and the 1000 top-ranked complexes were visually inspected. In compound selection, we considered interactions with key residues of the binding site, chemical diversity, and energy contributions to the binding energy that are not part of the scoring function (primarily ligand strain and binding site desolvation). None of the selected molecules contained motifs present in pan assay interference compounds.^38^ In total, 59 fragments (**F1**-**F59**) and 59 lead-like (**L1**-**L59**) compounds (Table 1 and Supporting Information Table S1) were purchased from commercial vendors.

**Table 1:**
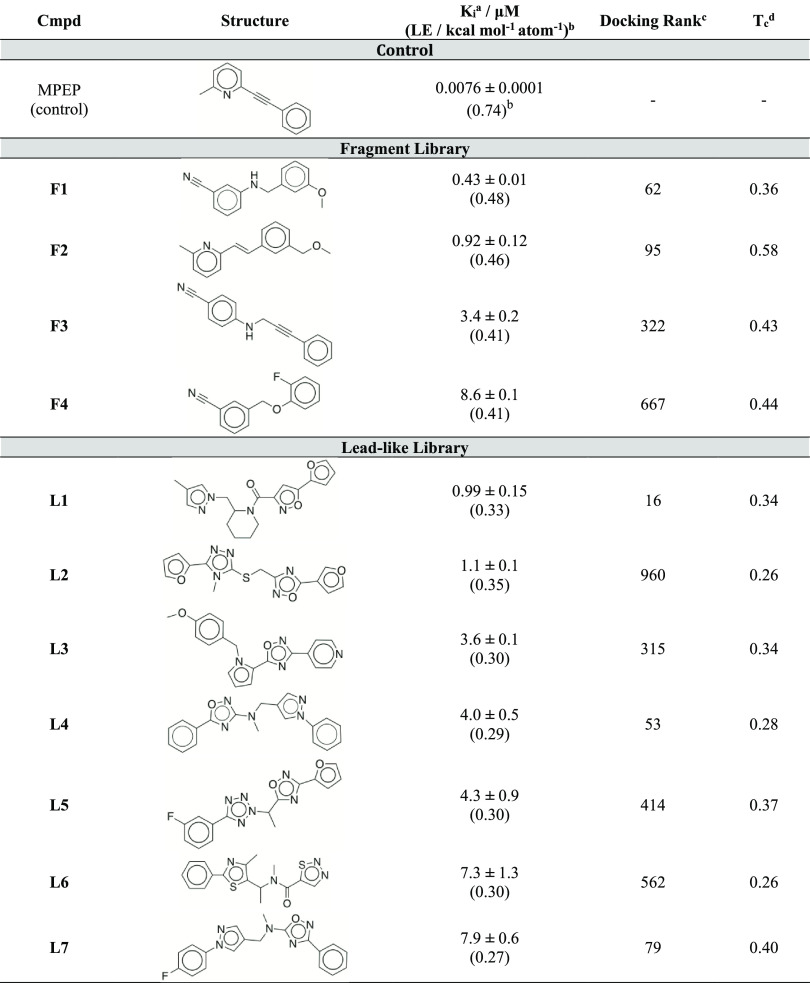
Structures and Experimental Binding Affinities of Ligands Discovered from the Virtual Screen

a Binding affinities were determined from radioligand displacement assays. Data represent mean values ± SEM of two experiments.

b Ligand efficiency (LE, kcal mol^–1^ heavy atom^–1^) was calculated as −*RT* ln(*K*_i_)/*N*. *K*_i_ and *N* are the binding affinity and number of ligand heavy atoms, respectively.^39^

c Ranking in the structure-based virtual screen of the ZINC12 fragment- or lead-like library.^40^

d Maximal Tanimoto similarity coefficient (*T*_c_) between the compound and all ChEMBL ligands of mGlu_5_ with a pChEMBL activity ≥5 (3188 compounds, ChEMBL28). *T*_c_ was calculated using RDKit with ECFP4 Fingerprints^41^ (1024 bits).

### Experimental Evaluation of Predicted Ligands

**Figure 3:**
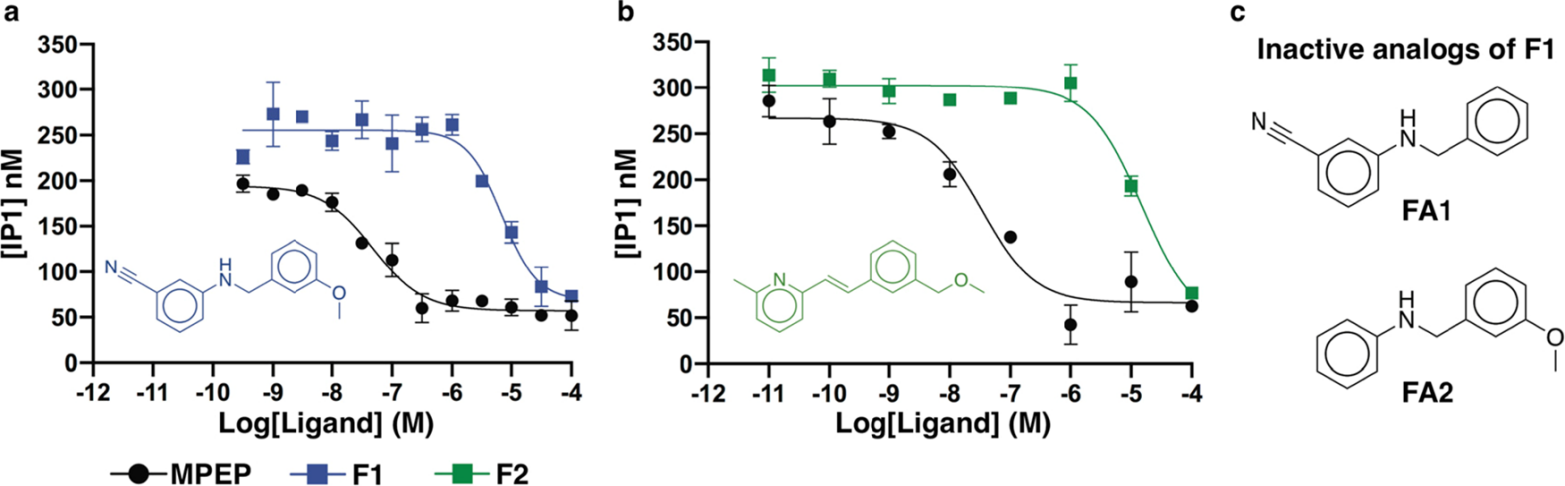
Evaluation of compounds **F1** and **F2** in functional assays. Representative dose–response curve of compounds (a) **F1** and (b) **F2** in an IP1 functional assay. Cells expressing the mGlu_5_ receptor were stimulated with a quisqualate concentration of 50 nM and a series of concentrations of **F1** (blue curve, pIC_50_ = 5.20 ± 0.11, *n* = 3) and **F2** (green curve, 5.10 ± 0.13 *n* = 5). Each point was performed in triplicate and is shown as a mean ± SEM of 3–5 independent experiments. The pIC_50_ of the reference NAM (MPEP) was determined to be 7.18 ± 0.13 in this assay (*n* = 5). (c) **FA1** and **FA2** are inactive analogues of **F1**.

The 118 predicted ligands were first tested in radioligand displacement assays at a concentration of 30 μM. Four fragments (**F1**-**F4**, Table 1) and nine lead-like compounds (**L1**-**L9**, Table 1 and Supporting Information Table S1) showed significant displacement of radiolabeled MPEP, a high-affinity NAM of mGlu_5_, and *K*_i_ values were determined for these ligands. The four fragments had binding affinities ranging from 0.43 μM (**F1**) to 8.6 μM (**F4**). The lead-like ligands showed *K*_i_ values between 0.99 μM (**L1**) and 16 μM (**L9**), and seven compounds had affinities better than 10 μM. The four compounds with the highest affinities (**F1**, **F2**, **L1**, and **L2**) were evaluated in a cell-based G protein assay measuring IP1 production induced by the agonist quisqualate and activation of G_q_ proteins. Compounds **F1** and **F2**, which originated from the fragment library, acted as NAMs in this assay with pIC_50_ values of 6.3 and 7.9 μM, respectively (Figure 3). The two lead-like ligands (**L1** and **L2**) also negatively modulated agonist-induced mGlu_5_-dependent IP1 production, but IC_50_ values could not be determined because of the low potency of these compounds (Supporting Information Figure S2). Two analogues of compound **F1** were also evaluated (**FA1** and **FA2**, Figure 3c) to assess the role of the nitrile- and methoxy-substituents. Both the analogues were inactive (IC_50_ > 100 μM), which is consistent with that both substituents of **L1** interact with the receptor in the predicted binding mode and that the ligand binds in an enclosed pocket.

**Figure 4:**
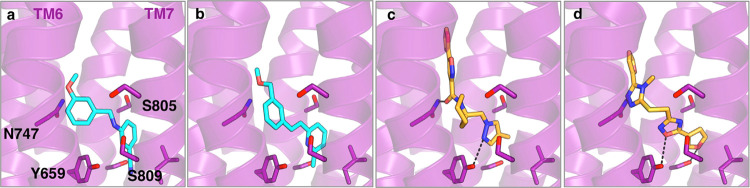
Predicted binding modes of allosteric ligands. Predicted binding modes of compounds (a) **F1**, (b) **F2**, (c) **L1**, and (d) **L2** in the allosteric binding pocket. The receptor is shown as a purple cartoon with key residues in sticks. The virtual screening hits are shown as sticks with either cyan (fragments **F1**-**F2**) or orange (lead-like compounds **L1**-**L2**) carbon atoms.

To assess the level of productive interactions established between ligand and receptor, we calculated the ligand efficiency (LE, i.e. the binding free energy per heavy atom, defined as −*RT* ln(*K*_i_)/*N*. *K*_i_ is the binding affinity and *N* is the number of ligand heavy atoms (HAs)) for the 11 compounds with *K*_i_ values better than 10 μM.^39^ The fragment ligands had LE values between 0.41 and 0.48 kcal mol^–1^ HA^–1^, whereas the LE values of the lead-like ligands ranged from 0.27 to 0.35 kcal mol^–1^ HA^–1^. The novelty of the identified ligands was assessed by comparing their 2D structures to previously identified mGlu_5_ ligands from the ChEMBL database,^42^ which contained 3188 molecules with activity <10 μM. The similarity was assessed by calculating the pair-wise Tanimoto coefficient (*T*_c_) using topological fingerprints, which ranges from 0 (no similarity between compound pair) to 1 (identical compounds). The *T*_c_ values of the four fragments ranged from 0.36 to 0.58, and the most novel of these had the highest affinity (compound **F1**, *K*_i_ = 0.43 μM). The fragments were composed of two aromatic rings connected by a short linker moiety, which is a feature present in several NAMs (Figure 2). As reflected by the relatively high *T*_c_ values, compounds **F2**-**F4** were similar to previously identified NAMs (Supporting Information Table S2). We do not consider these fragments to represent novel scaffolds, but it is encouraging that docking was able to identify compounds that are dissimilar to the cocrystallized ligand (Mavoglurant), demonstrating the strength of a structure-based approach. The novel linker exemplified by **F1** could be an attractive alternative to the acetylene present in several NAMs (Figure 2) as this moiety can have unfavorable ADME properties.^43^ Among the seven lead-like compounds, *T*_c_ values ranged between 0.26 and 0.40, and several of the compounds represented novel ligand chemotypes (Supporting Information Table S2). The fragment-like hits primarily overlapped with the pockets occupied by Mavoglurant, whereas the lead-like compounds also extended into other subpockets (Figure 4 and Supporting Information Figure S3).

### Comparison of Screens Using Fragment- and Lead-Like Chemical Libraries

Comparisons of the results from the two screens illustrated distinct advantages of each screening library. On the one hand, the hit rate (*K*_i_ < 10 μM) from the lead-like library (12%) was slightly higher than from the fragment-like library (7%). This could reflect that there are likely fewer ligands in the fragment library with *K*_i_ values lower than 10 μM, as these can form fewer interactions with the binding site. On the other hand, the fragments consistently had better LE values and would hence be considered to represent better starting points for hit-to-lead optimization.^44^ The lead-like library clearly resulted in more novel scaffolds. Based on Tanimoto similarity, the five most novel ligands were from the lead-like library, and only one of the fragment ligands had a *T*_c_ value <0.4. In this context, one may also ask what the docking result would be if the fragment- and lead-like compounds were combined into a single screening library. The lead-like compounds that were considered for experimental testing had docking scores better than −43 kcal/mol, whereas the top-ranked fragment had a predicted binding energy of −36.5 kcal/mol. As docking scoring functions generally favor larger compounds,^45^ none of the fragment ligands would have been present among the top-ranked molecules in the lead-like library and hence not be considered for testing (Supporting Information Figure S4). Clearly, as demonstrated by that the highest affinity ligand was a fragment, the-bigger-the-better bias of docking scoring functions is a flaw that can lead to hits with poor ligand efficiency. Another interesting observation is that the chemotypes identified from the fragment- and lead-like libraries were dissimilar, and the fragment hits were not substructures of the lead-like ligands. Whereas the hits from the fragment library were all composed of two six-membered aromatic rings, the lead-like ligands were more diverse and also contained several different five-membered heteroaromatic rings. Similarly, the predicted binding modes of the compounds with the highest affinities suggested that key interactions were formed by different chemical groups for the fragments and lead-like ligands (Figure 4). The two best fragments occupied the hydrophobic pocket formed by TM helices 3–5 with six-membered aromatic rings, whereas five-membered aromatic rings interacted with the same site for the lead-like ligands. Access to several classes of ligands that form different interactions in the same binding site can be valuable in hit-to-lead optimization. The fact that the two screens showed complementary advantages and resulted in the discovery of different scaffolds suggests that the optimal strategy is to carry out parallel virtual screens of fragment- and lead-like libraries.

Both the fragment- and lead-like hits antagonized mGlu_5_ in a functional assay, which is consistent with that the virtual screen was performed using a crystal structure determined in complex with a NAM. This result was unexpected considering that small structural modifications of mGlu_5_ ligands can transform a NAM into a PAM.^46^ Several new crystal and cryo-EM structures of mGlu_5_ have recently been determined,^8,9,17,19,20^ which in combination with computational modeling provided new insights into the mechanism of allosteric modulation. Structures of complexes with diverse NAMs show that compounds stabilize different conformations of the allosteric pocket and that perturbation of hydrogen bonding networks lead to different functional effects.^47,48^ Accounting for such induced-fit effects and water-mediated ligand interactions will be important in optimization of the affinity and functional effect of allosteric modulators. Although the more recently published mGlu_5_ structures did not show significantly improved ability to enrich known ligands (Supporting Information Table S3), docking screens using alternative conformations of the pocket could facilitate identification of novel ligand chemotypes with NAM or PAM activity.

In contrast to lead discovery targeting the orthosteric site of GPCRs, allosteric modulators cannot be designed based on the structure of the endogenous ligand. A large number of mGlu allosteric modulators have instead been identified by high-throughput screening (HTS) of chemical libraries.^19,49−57^ Our results show that structure-based virtual screens can complement empirical screening. The overall virtual screening hit rate compares favorably to HTS campaigns to identify mGlu_5_ modulators. For example, Rodriguez et al. screened 160,000 compounds using functional assays and identified 345 NAMs of mGlu_5_, corresponding to a hit rate of 0.2%.^57^ Our docking approach allowed us to explore a larger library with several million lead-like compounds but only involved experimental testing of 59 compounds and resulted in a >50-fold higher hit rate. Fragment-based screening has also been applied successfully to identify mGlu_5_ NAMs. Christopher et al. screened 3600 fragments by radioligand binding, resulting in 178 hits (5% of the fragments showed >30% displacement at 300 μM) and one of the most promising NAMs showed an affinity of 2.5 μM (LE = 0.36).^19^ We identified fragments with comparable affinities and obtained a higher hit rate (7% of the tested fragments had affinities <10 μM, and 37% showed >30% inhibition at 30 μM).

### Structure-Guided Discovery of Ligands Binding to Allosteric Sites of GPCRs

Structure-based virtual screening has identified ligands binding to the orthosteric pocket of class A GPCRs,^6^ but can the same success rates be expected for allosteric sites? The NAM binding site of mGlu_5_ shares many similarities to the orthosteric site of class A GPCRs that bind small molecules, for example, monoamine and adenosine receptors. The pocket is small and buried in the TM region, and the ligands form polar interactions with a few key residues, resulting in a druggable site that binds small molecules with high affinity. Most of the other allosteric sites of GPCRs that have been identified by structural biology do not have such features. Rather, the allosteric sites identified in class A GPCRs (e.g., the M_2_ muscarinic acetylcholine, Protease-activated receptor 2, and free fatty acid 1 receptors) are less well-defined and are either solvent-exposed or located in extrahelical sites facing the membrane.^6^ Molecular docking to such sites can be expected to be challenging and, in agreement with these observations, virtual screening hit rates have been lower in these cases compared to orthosteric sites.^6,58^ These observations suggest that assessing the druggability of potential binding sites is a crucial step for successful application of structure-based screening for allosteric modulators.

## Conclusions

In this work, structure-based virtual screens against an allosteric binding pocket of mGlu_5_ were performed. Eleven allosteric ligands were identified and the most potent also antagonized mGlu_5_ activity in functional assays. Parallel docking screens of fragment- and lead-like chemical libraries yielded similar hit rates but identified ligands with complementary advantages in terms of novelty, physicochemical properties, and interactions with the receptor. Our results demonstrate that docking screens of chemical libraries can contribute to the discovery of ligands with an allosteric mode of action, which could lead to the development of a new generation of GPCR drugs.

## Methods

### Molecular Docking Screens

The molecular docking calculations were performed with DOCK3.6^31^ using a crystal structure of mGlu_5_ in complex with the NAM Mavoglurant (PDB accession code: 4OO9).^9^ In preparation of the structure for docking, all nonprotein atoms and the T4-lysozyme fusion were removed. The allosteric binding site contained one crystallographic water molecule. As a previous study demonstrated that including water molecules in the docking calculations did not improve ligand enrichment,^47^ the virtual screen was performed without water molecules in the binding pocket. The side chains of the ionizable residues Lys, Glu, Asp, and Arg were modeled to represent their most probable protonation state at pH 7.4. The binding site was defined by the position of the cocrystallized ligand. The flexible ligand sampling algorithm of DOCK3.6 was used to dock the compounds to the allosteric site based on 45 matching spheres, which represent putative ligand atom positions, with a matching tolerance of 1.5 Å, bin overlap of 0.3 Å, and bin sizes of 0.4 Å. Chemical matching was used on the matching spheres based on their local receptor environment.^59^ The DOCK3.6 scoring function predicts the binding energy as the sum of the electrostatic and van der Waals binding energies, corrected for ligand desolvation.^31,32^ The scoring grids for these energy terms were calculated using DOCK3.6. A rigid-body energy minimization (100 steps) was carried out for the best scoring conformation of each docked compound. The enrichment of ligands was evaluated based on docking of 212 mGlu_5_ NAMs and 9399 property-matched decoys. In the prospective screen, two receptor models with different rotamers for the polar hydrogen of Ser809^7.39x40^ were used. The ZINC12 fragment library (1.6 million compounds, *M*W ≤ 250 Da) and lead-like library (4.6 million compounds, *M*W = 250–350 Da) were docked to the allosteric pocket.^37^ For each library, the results were combined into a single ranked list based on the docking scores. Retrospective molecular docking calculations for several mGlu_5_ structures (PDB accession codes: 4OO9,^9^ 7P2L,^17^ 5CGD,^19^ 6FFI,^20^ and 6FFH^20^) were prepared and performed with DOCK3.7 protocols.^60^ Tanimoto similarity coefficients were calculated using RDKit (version 2017.03.2) with ECFP4 Fingerprints^41^ of 1024 bits.

### Radioligand Displacement Assays

The 118 selected compounds were purchased from commercial vendors (Table 1 and Supporting Information Table S1, vendor purity >90%). Assessment of compound purity by LC/MS for hits from the screen showed that the first sample of **F2** had low purity (53%). **F2** was then resynthesized, and new binding assays confirmed the affinity of the pure sample (*K*_i_ = 0.55 μM), which was used in functional assays. HEK293 cells, which stably expressed mGlu_5_ receptor, were treated with sodium butyrate (10 mM final concentration in growth media) for 24 h prior to harvest. Treated cells were harvested with PBS/2 mM EDTA and washed three times with ice-cold PBS and frozen at −80 °C. The frozen cell pellet was resuspended in membrane buffer (25 mM Tris/7.4, 250 mM sucrose, 2.5 mM EDTA, 2 ug/ml aprotinin, 0.5 ug/ml leupeptin and 200 nM PMSF) and homogenized with a polytron 20–30 s at maximum power. After centrifugation at 18,000 RPM for 30 min at 4 °C, the pellet was resuspended in ice-cold membrane buffer, hand-homogenized, and centrifuged as described above. This pellet was further resuspended in ice-cold membrane buffer. The protein content was measured using the Bradford method with bovine serum albumin as the standard. The membrane homogenate was frozen at −80 °C before use. After thawing, the membranes were washed once and resuspended in ice-cold 50 mM Tris–HCl, 0.9% NaCl, pH 7.4 buffer. All incubations were performed at room temperature. For the displacement binding experiments, 15 μg of membranes were incubated with 5 nM the radioligand in the presence of 10 varying concentrations of the test compound for 2 h at room temperature with shaking. At the end of the respective incubations, the suspension was filtered onto PerkinElmer GF/C glass fiber filters (1450–421) pre-soaked in 0.5% polyethyleneimine and washed rapidly four times using a Tomtec Harvester 96 Mach III cell harvester (Tomtec, Hamden, CT) with 5 mL of cold wash buffer (50 mM Tris–HCl, pH 7.4). The radioactivity trapped on the filters was measured after heat sealing the filters with MeltiLexTM A (PerkinElmer, 1450–441) in a 1450 MicroBeta TriLux counter (PerkinElmer). Nonspecific binding was defined in the presence of 10 μM MPEP. IC_50_ values were derived from the inhibition curve, and *K*_i_ values were calculated according to the Cheng-Prusoff equation of *K*_i_ = IC_50_/(1 + [*L*]/*K*_d_),^61^ where [*L*] is the concentration of radioligand and *K*_d_ is its dissociation constant at the receptor, derived from the saturation isotherm.

### IP1 Functional Assay

HEK293 cells were cultured in Dulbecco’s modified Eagle’s medium (DMEM) supplemented with 10% fetal bovine serum (FBS) and were maintained at 37 °C in a humidified atmosphere with 5% CO_2_. Transient transfection was performed using electroporation in a volume of 200 mL with 0.6 mg of plasmid encoding SNAP-tag human mGlu_5_, 2 mg plasmid encoding for the glutamate transporter EAAC1 plasmids, and 10 million of HEK293 cells in electroporation buffer (50 mM K_2_HPO_4_, 20 mM CH_3_COOK, and 20 mM KOH, pH 7.4). After electroporation (250 V, 0.5 mF, Bio–Rad Gene Pulser electroporator; Bio-Rad Laboratories, Hercules, CA), cells were resuspended in 10 mL of DMEM supplemented with 10% FBS and seeded for 24 h into black, clear-bottom 96-well culture plates (Greiner Bio-one), pretreated with Poly-l-Ornithine 1X, at a density of 100,000 cells per well. Following 24 h of transfection, HEK293 cells were incubated for 2 h with glutamate-free DMEM GlutaMAX-I (Life Technologies). The IP1 accumulation assay kit (Cisbio Bioassays, PerkinElmer) was used for the direct quantitative measurement of IP1. Cells were stimulated by quisqualate at EC_80_ alone and with various concentrations of allosteric compounds, incubated for 30 min at 37 °C, 5% CO_2_. Cells were then lysed using the conjugate-lysis buffer mixed with the d2-labeled IP1 analogue and the Lumi4-terbium cryptate-labeled anti-IP1 antibody according to the manufacturer’s instructions. After a 1 h incubation at room temperature, the HTRF measurement was performed after excitation at 337 nm with 50 μs delay. Terbium cryptate fluorescence and tr-FRET signals were measured at 620 and 665 nm, respectively, using a PheraStar fluorimeter (BMG Labtech).

## Supporting Information Available

Supporting data from the molecular docking screens, Tanimoto similarity calculations, and experimental assays (PDF)
