# Supplementary material for: Structure-Based Discovery of Negative Allosteric Modulators of the Metabotropic Glutamate Receptor 5
Source: ACS Chem Biol. 2022 Sep 23;17(10):2744–52. doi: 10.1021/acschembio.2c00234 (PMC9594040; doi:10.1021/acschembio.2c00234)
Supplement: Supplementary file 1 — cb2c00234_si_001.pdf [file cb2c00234_si_001.pdf]

Supporting Information:

Structure-Based Discovery of Negative Allosteric Modulators of the Metabotropic Glutamate Receptor 5

Stefanie Kampen[1,†], David Rodríguez[2,3,6,†], Morten Jørgensen[3,7], Monika Kruszyk-Kujawa[3,8], Xinyan Huang[4,9], Michael Collins Jr.[4,10], Noel Boyle[4,11], Damien Maurel[5], Axel Rudling[2], Guillaume Lebon[5], and Jens Carlsson[1,*]

[1] Science for Life Laboratory, Department of Cell and Molecular Biology, Uppsala University, SE-751 24 Uppsala, Sweden

[2] Science for Life Laboratory, Department of Biochemistry and Biophysics, Stockholm University, SE-171 21 Solna, Sweden

[3] H. Lundbeck A/S, Ottiliavej 9, DK-2500 Valby, Denmark

[4] Lundbeck Research USA, 215 College Road, Paramus, NJ 07652 - 1431, United States

[5] IGF, Université de Montpellier, CNRS, INSERM, 34094 Montpellier, France

[6] Present address: Novo Nordisk, DK 2760 Maaloev, Denmark

[7] Present address: LEO Pharma, Drug Design, Industriparken 55, DK-2750 Ballerup, Denmark

[8] Present address: Selvita Poznań WCZT, ul. Uniwersytetu Poznańskiego 10 61-614, Poznań, Poland

[9] Present address: NYU Langone Health, One Park Ave, New York, NY 10016, United States

[10] Present address: Pfizer, 401 North Middletown Rd, Pearl River, NY 10965, United States

[11] Present address: 412 Ridge Drive, Union, NJ 07083, United States

*E-mail: jens.carlsson@icm.uu.se

[†]Contributed equally to this work

Table of contents

	Page
**Tables**	
**Table S1**. Compounds selected from the virtual screen	S2
**Table S2.** Most similar known $mGlu_5$ ligands from the ChEMBL database	S10
**Table S3.** Enrichment of NAMs by different $mGlu_5$ structures	S11
**Figures**	
**Figure S1.** Enrichment of $mGlu_5$ allosteric modulators by the crystal structure	S11
**Figure S2.** Functional assay for compounds **L1** and **L2**	S12
**Figure S3.** Predicted binding modes of compounds **F3-F4** and **L3-L7**	S13
**Figure S4.** Distributions of docking scores for the libraries	S14
**References**	S15

Tables

Table S1. Compounds selected from the virtual screen of fragment- and lead-like libraries.

Cmpd	Structure	K_i[a] (µM) or Inhibition at 30 µM (%)[a]	Docking Rank[b]	Similarity to $mGlu_5$ Ligands[c]
		**Fragment library**		
**F5**	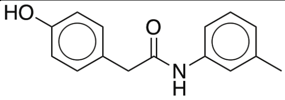	42 ± 5%	893	0.40
**F6**	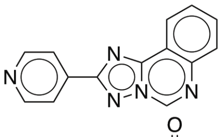	40 ± 3%	705	0.29
**F7**	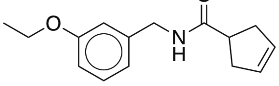	39 ± 7%	656	0.30
**F8**	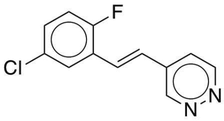	39 ± 1%	854	0.26
**F9**	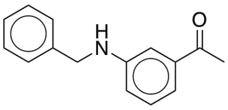	38 ± 1%	695	0.31
**F10**	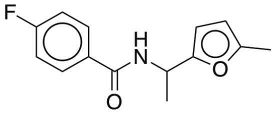	35 ± 8%	930	0.35
**F11**	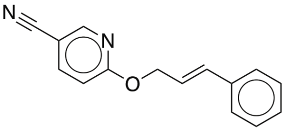	35 ± 6%	5	0.33
**F12**	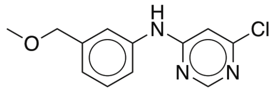	35 ± 10%	28	0.31
**F13**	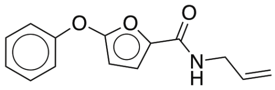	35 ± 10%	312	0.29
**F14**	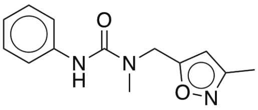	33 ± 4%	418	0.32
**F15**	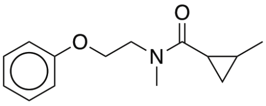	33 ± 9%	969	0.33
**F16**	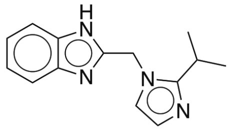	33 ± 5%	229	0.22
**F17**	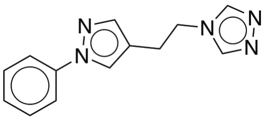	33 ± 3%	74	0.27
**F18**	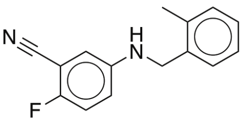	33 ± 9%	746	0.28

**F19**	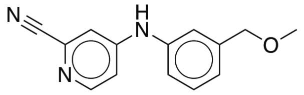	32 ± 4%	859	0.36
**F20**	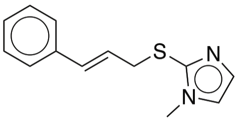	31 ± 1%	334	0.35
**F21**	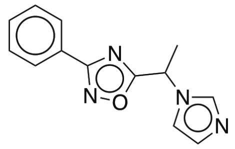	31 ± 1%	319	0.34
**F22**	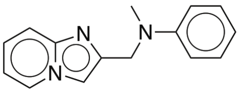	31 ± 1%	128	0.39
**F23**	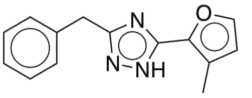	30 ± 7%	359	0.27
**F24**	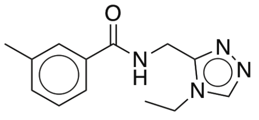	30 ± 1%	810	0.40
**F25**	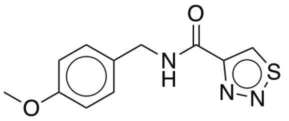	28 ± 9%	937	0.31
**F26**	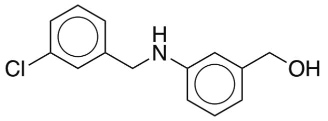	28 ± 15%	38	0.38
**F27**	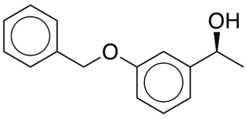	27 ± 3%	693	0.45
**F28**	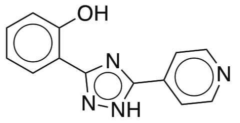	27 ± 3%	521	0.29
**F29**	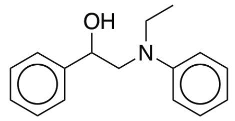	27 ± 1%	176	0.25
**F30**	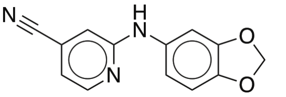	27 ± 3%	537	0.31
**F31**	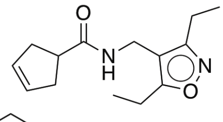	27 ± 1%	110	0.22
**F32**	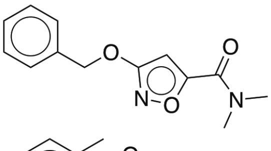	27 ± 1%	801	0.39
**F33**	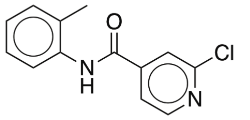	26 ± 11%	784	0.36
**F34**	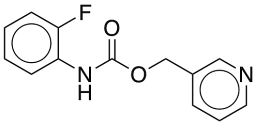	26 ± 4%	451	0.34

**F35**	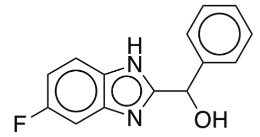	25 ± 12%	315	0.23
**F36**	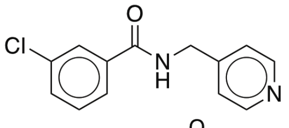	25 ± 3%	24	0.51
**F37**	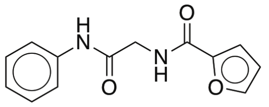	25 ± 4%	836	0.36
**F38**	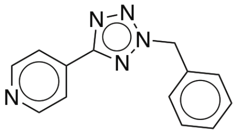	25 ± 3%	112	0.35
**F39**	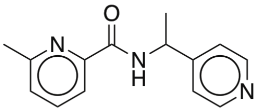	24 ± 9%	729	0.54
**F40**	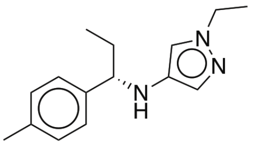	24 ± 7%	978	0.23
**F41**	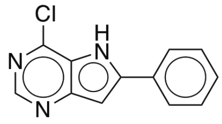	24 ± 2%	572	0.25
**F42**	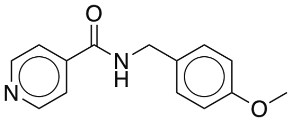	24 ± 8%	395	0.32
**F43**	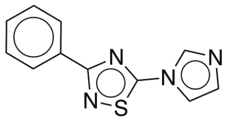	23 ± 2%	665	0.26
**F44**	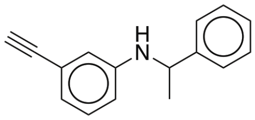	23 ± 4%	830	0.28
**F45**	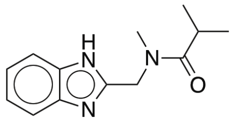	22 ± 9%	844	0.23
**F46**	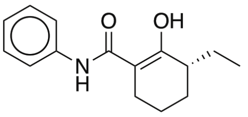	22 ± 1%	419	0.34
**F47**	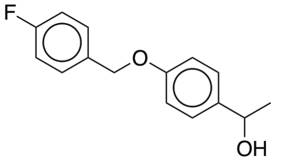	21 ± 3%	371	0.37
**F48**	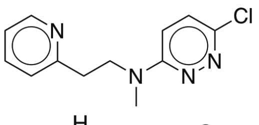	21 ± 1%	503	0.29
**F49**	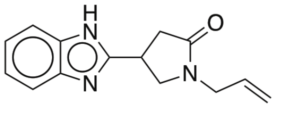	20 ± 1%	77	0.29

**F50**	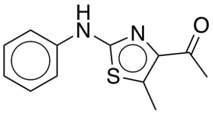	20 ± 9%	898	0.31
**F51**	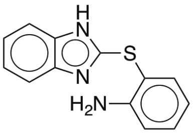	20 ± 14%	657	0.21
**F52**	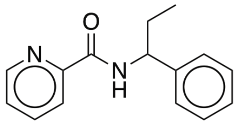	19 ± 5%	488	0.46
**F53**	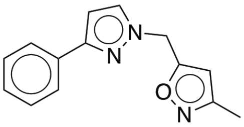	19 ± 2%	880	0.30
**F54**	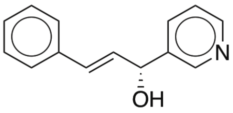	19 ± 3%	804	0.32
**F55**	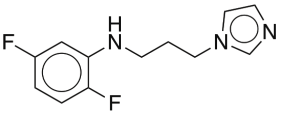	19 ± 5%	114	0.26
**F56**	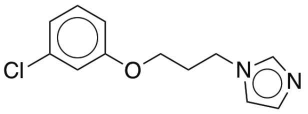	18 ± 4%	139	0.35
**F57**	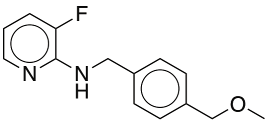	17 ± 3%	833	0.33
**F58**	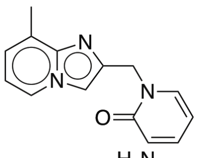	16 ± 5%	529	0.27
**F59**	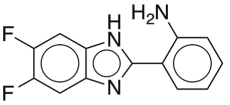	9 ± 1%	296	0.22
**Lead-like library**				
**L8**	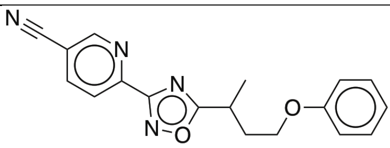	11 ± 1	146	0.46
**L9**	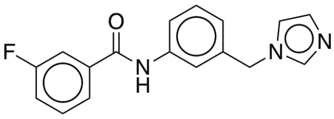	16 ± 3	248	0.40
**L10**	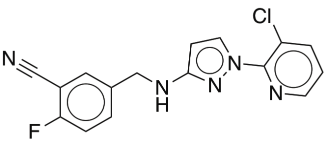	41 ± 7%	460	0.32
**L11**	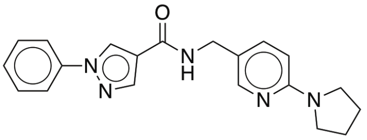	40 ± 4%	139	0.31

**L12**	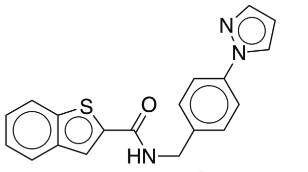	39 ± 6%	341	0.26
**L13**	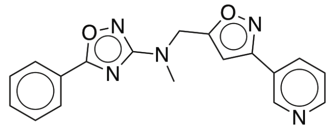	39 ± 4%	436	0.34
**L14**	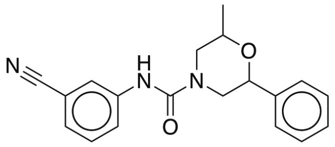	38 ± 12%	362	0.41
**L15**	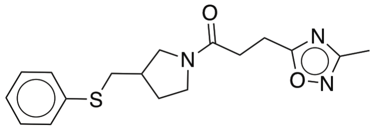	37 ± 6%	620	0.32
**L16**	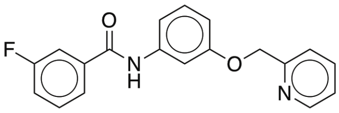	36 ± 1%	570	0.44
**L17**	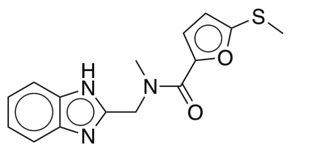	35 ± 3%	308	0.23
**L18**	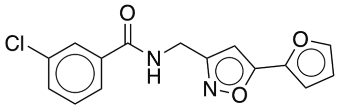	34 ± 1%	730	0.40
**L19**	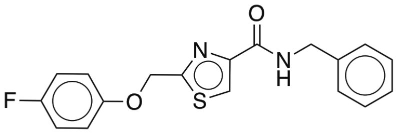	34 ± 3%	6	0.37
**L20**	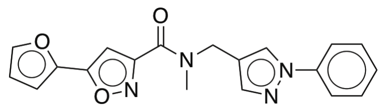	34 ± 5%	76	0.24
**L21**	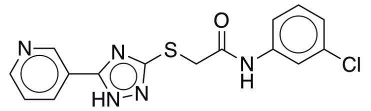	33 ± 12%	532	0.40
**L22**	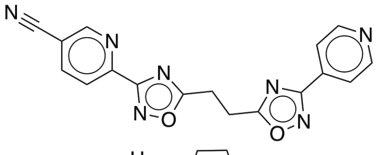	33 ± 1%	505	0.50
**L23**	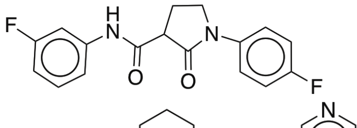	32 ± 1%	351	0.41
**L24**	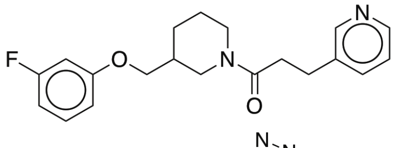	32 ± 3%	448	0.38
**L25**	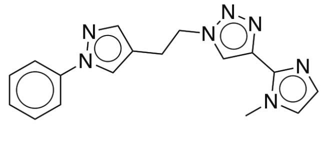	32 ± 4%	991	0.29

**L26**	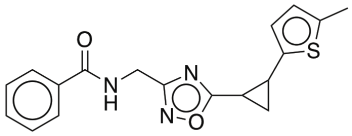	31 ± 7%	157	0.29
**L27**	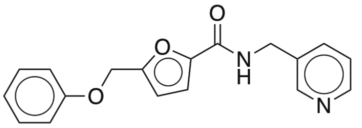	31 ± 7%	135	0.34
**L28**	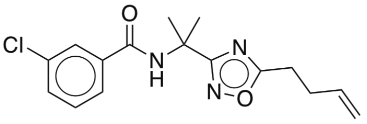	31 ± 13%	548	0.36
**L29**	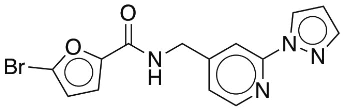	30 ± 9%	591	0.31
**L30**	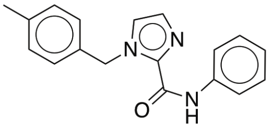	30 ± 5%	735	0.36
**L31**	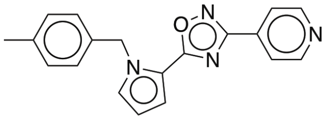	29 ± 4%	201	0.31
**L32**	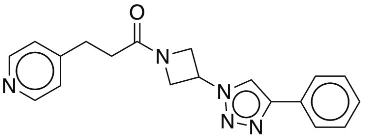	29 ± 3%	767	0.35
**L33**	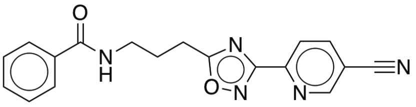	29 ± 6%	84	0.43
**L34**	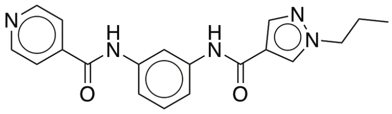	29 ± 1%	340	0.34
**L35**	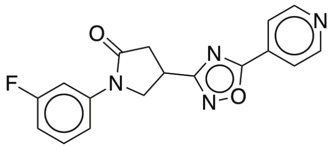	29 ± 1%	209	0.50
**L36**	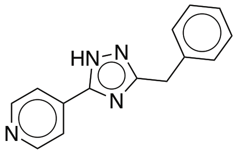	29 ± 8%	666	0.28
**L37**	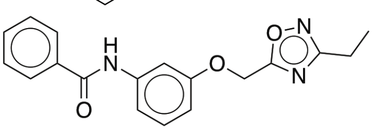	29 ± 4%	919	0.38
**L38**	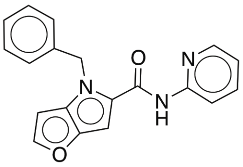	27 ± 1%	302	0.40
**L39**	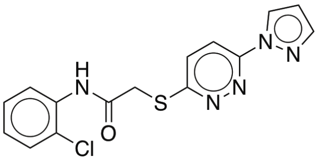	27 ± 11%	154	0.34

**L40**	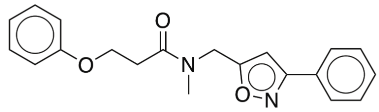	27 ± 3%	116	0.31
**L41**	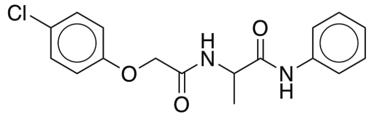	26 ± 2%	649	0.33
**L42**	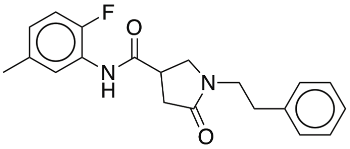	26 ± 5%	187	0.32
**L43**	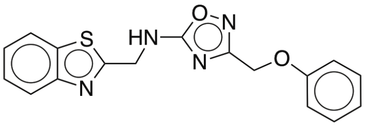	26 ± 12%	150	0.34
**L44**	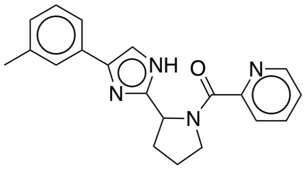	26 ± 13%	239	0.58
**L45**	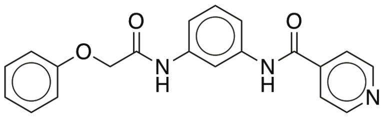	25 ± 7%	2	0.40
**L46**	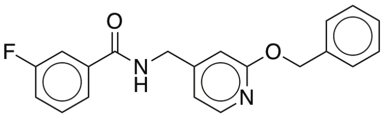	24 ± 3%	11	0.41
**L47**	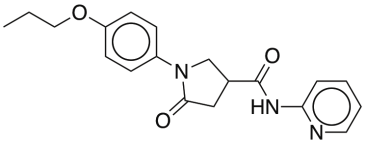	24 ± 3%	820	0.34
**L48**	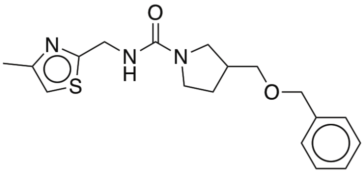	23 ± 4%	608	0.35
**L49**	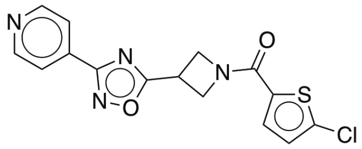	22 ± 1%	346	0.51
**L50**	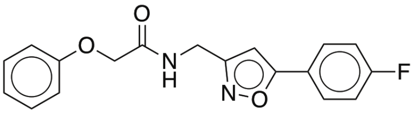	20 ± 1%	831	0.35
**L51**	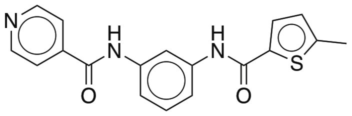	20 ± 6%	52	0.38
**L52**	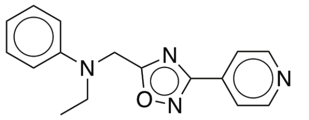	20 ± 1%	990	0.29
**L53**	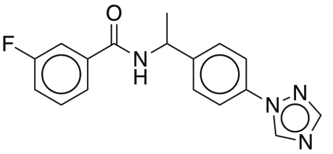	19 ± 2%	736	0.40

**L54**	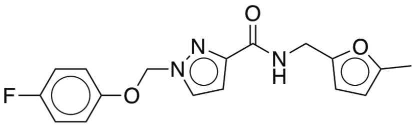	18 ± 3%	45	0.30
**L55**	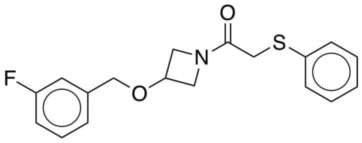	18 ± 3%	102	0.38
**L56**	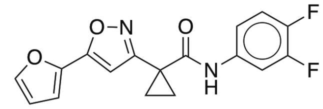	18 ± 6%	787	0.28
**L57**	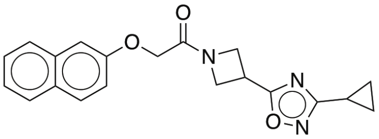	17 ± 2%	982	0.37
**L58**	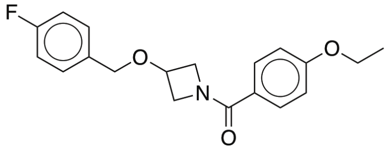	16 ± 2%	922	0.42
**L59**	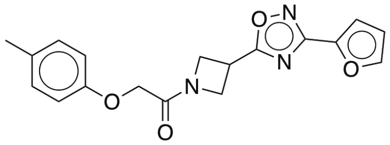	15 ± 1%	723	0.46

[a] Binding affinities were determined from radioligand displacement assays. Data represent mean values ± SEM of two experiments.
[b] Ranking in the structure-based virtual screen of the ZINC12 fragment- or lead-like library[1].
[c] Maximal Tanimoto coefficient (T_c) between the compound and all ChEMBL ligands of $mGlu_5$ with a pChEMBL activity ≥ 5 (3188 compounds, ChEMBL28). T_c was calculated using RDKit with ECFP4 Fingerprints (1024 bits).

Table S2. Most similar known mGlu$_5$ ligands from the ChEMBL database.

Cmpd	Structure	T_c[a]	ChEMBL ID	ChEMBL structure
**F1**	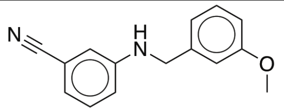	0.36	CHEMBL20444	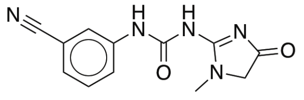
**F2**	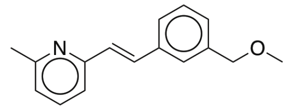	0.58	CHEMBL88612	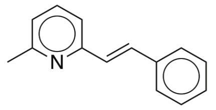
**F3**	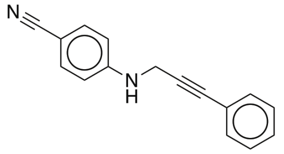	0.43	CHEMBL214823	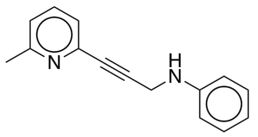
**F4**	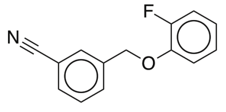	0.44	CHEMBL206579	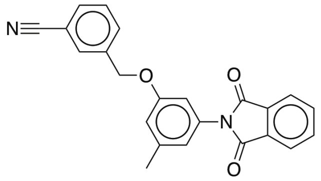
**L1**	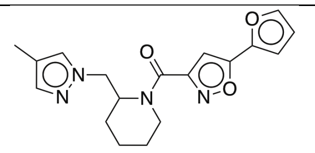	0.34	CHEMBL1771681	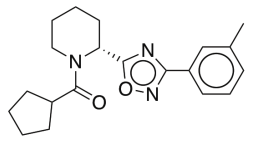
**L2**	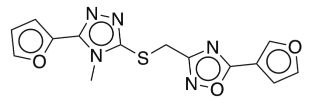	0.26	CHEMBL271065	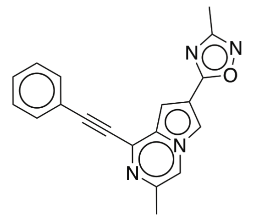
**L3**	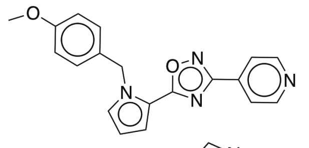	0.34	CHEMBL1487967	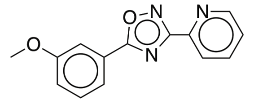
**L4**	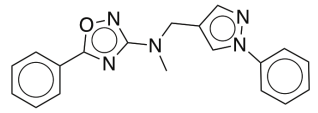	0.28	CHEMBL2164558	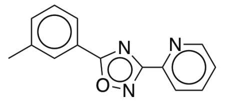
**L5**	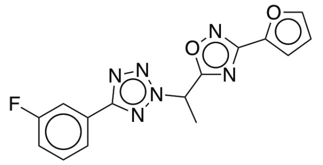	0.37	CHEMBL186864	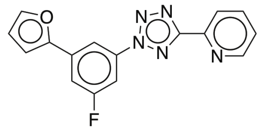
**L6**	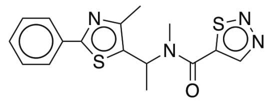	0.26	CHEMBL4548820	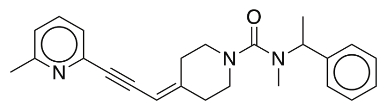
**L7**	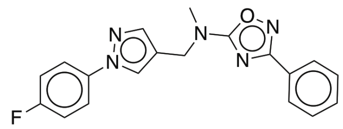	0.40	CHEMBL2071580	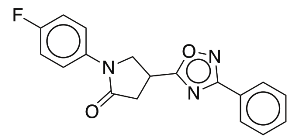

[a] Maximal Tanimoto similarity coefficient (T_c) between the compound and all ChEMBL ligands of mGlu$_5$ with a pChEMBL activity $\geq$ 5 (3188 compounds, ChEMBL28). T_c was calculated using RDKit with ECFP4 Fingerprints (1024 bits).

Table S3. Enrichment of NAMs by different $mGlu_5$ structures.

PDB Code	Ligand bound	LogAUC	EF_1
4OO9[a]	Mavoglurant	13	7
7P2L	Alloswitch-1	9	4
5CGD	HTL14242	6	3
6FFI	MMPEP	12	8
6FFH	Fenobam	12	9
All structures[b]	-	14	9

[a] The results for this receptor structure are different from those shown in Supporting Information Figure S1 because a different version of DOCK (3.7) and default parameters were used.

[b] Enrichments were calculated based on the best docking score for each compound using the results from all $mGlu_5$ structures (4OO9, 7P2L, 5CGD, 6FFI and 6FFH).

Figures

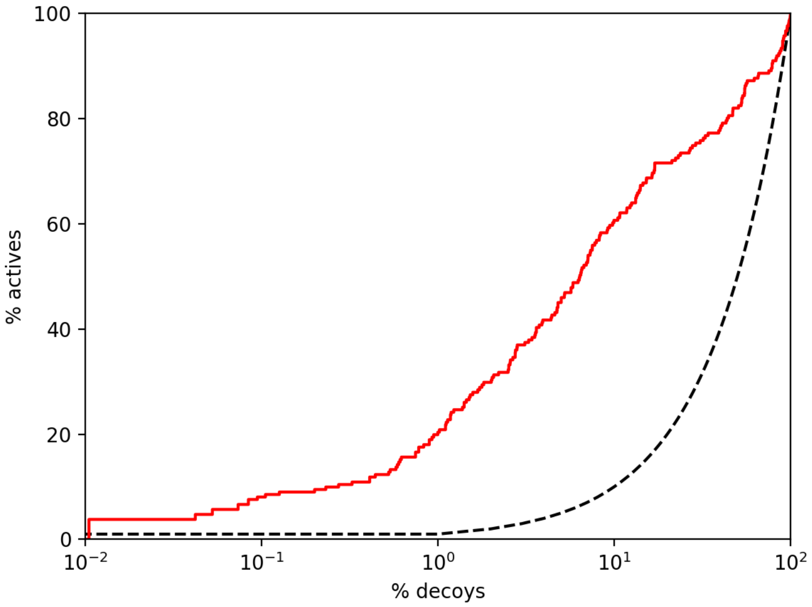

Figure S1. Enrichment of $mGlu_5$ allosteric modulators by the crystal structure. The enrichment of ligands by the structures used in virtual screening based on docking of $mGlu_5$ NAMs and property-matched decoys (red curve). The black dashed line represents the curve expected for random ligand enrichment.

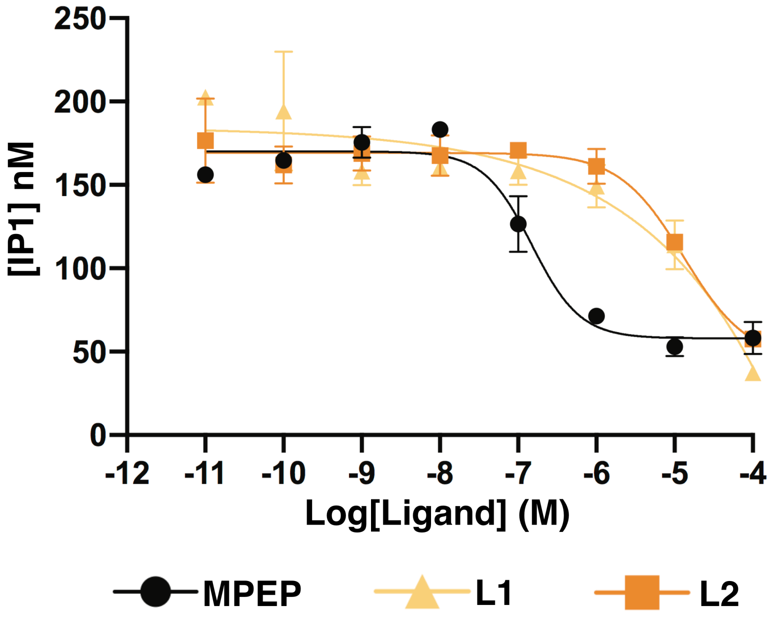

Figure S2. Functional assays for compounds L1 and L2. (a) Representative dose- response curve of compounds **L1** and **L2** in an IP1 functional assay. Cells expressing mGlu$_5$ receptor were stimulated with quisqualate at a concentration of 50 nM and a series of concentrations of **L1**, **L2,** and MPEP (reference NAM).

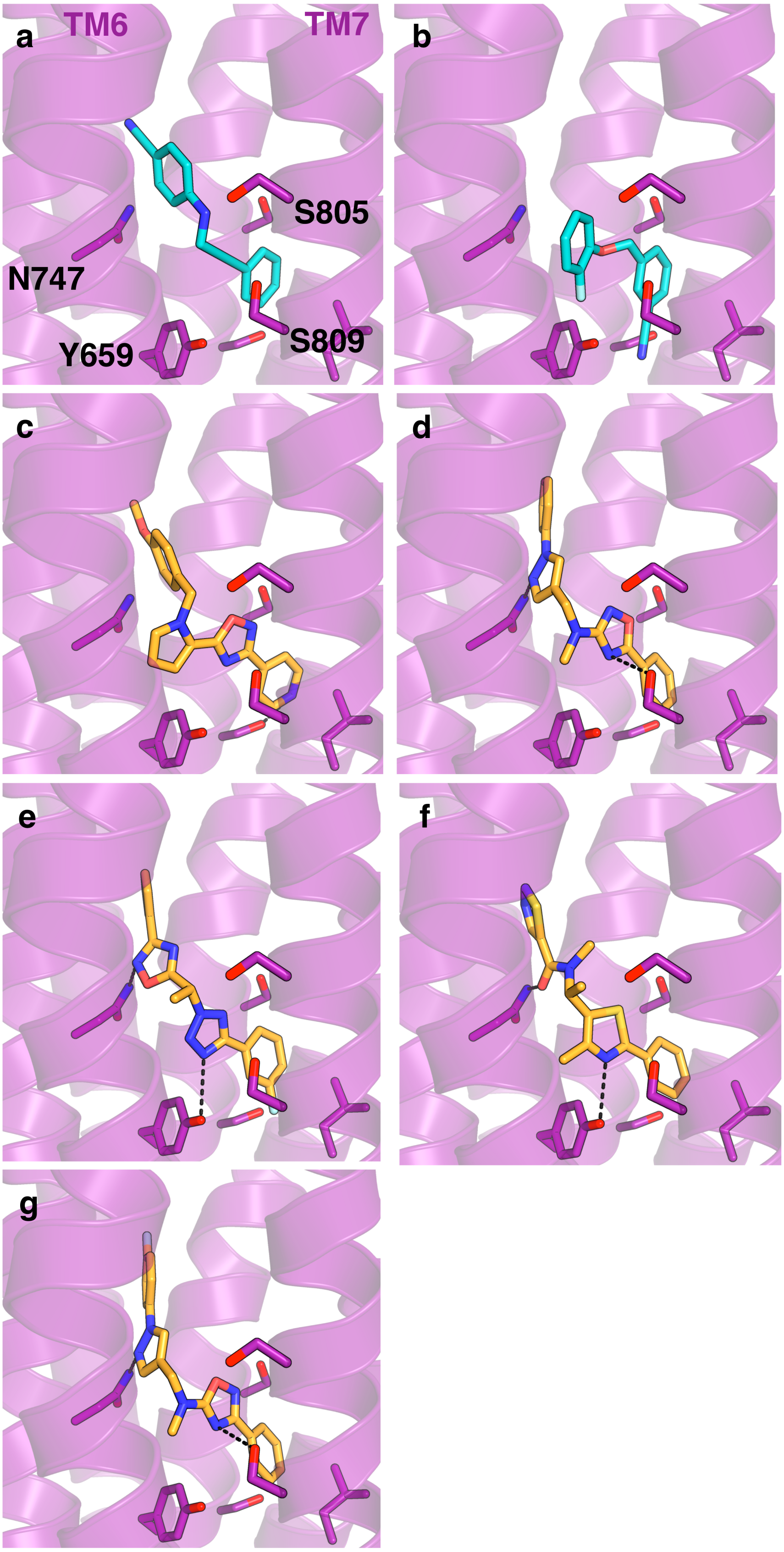

Figure S3. Predicted binding modes of compounds F3-F4 and L3-L7. (a) **F3**, (b) **F4**, (c) **L3**, (d) **L4**, (e) **L5**, (f) **L6**, and (g) **L7**. The receptor is shown as cartoons with key residues in sticks (purple). The virtual screening hits are shown as sticks with either cyan (fragments **F3-F4**) or orange (lead-like compounds **L3-L7**) carbon atoms.

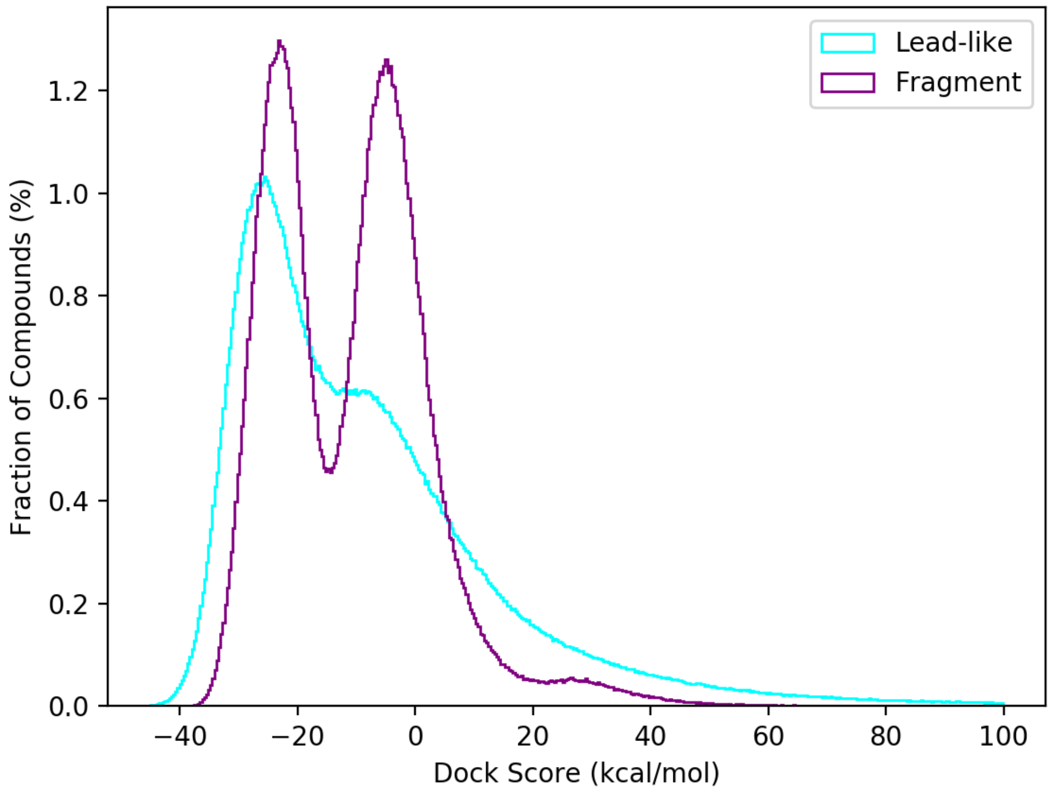

Figure S4. Distribution of docking scores for the fragment- and lead-like libraries. Distributions of docking scores for fragment- (purple) and lead-like compounds (cyan) docked to the mGlu$_5$ allosteric pocket.

References

(1) Sterling, T., Irwin, J. J. (2015) ZINC 15 - Ligand Discovery for Everyone. *J. Chem. Inf. Model.* 55, 2324–2337.
